# COVID-19-Associated Bilateral Avascular Necrosis of Femoral Head in a Young Male Without Corticosteroid Exposure: A Case Report

**DOI:** 10.7759/cureus.57525

**Published:** 2024-04-03

**Authors:** Lakshmi Murugesan, Nithila Sivakumar, Lakshiya Ramamoorthy, Umar Farooq

**Affiliations:** 1 Internal Medicine, Knights Medical Associates, Bensalem, USA

**Keywords:** ischemic hip necrosis, avascular femoral head necrosis, covid-19, bilateral avascular necrosis of hip, osteonecrosis

## Abstract

Avascular necrosis (AVN), also known as osteonecrosis, ischemic bone necrosis, or aseptic necrosis, is a progressive bone disease marked by the deterioration of bone tissue due to compromised blood flow in the subchondral region. AVN is typically caused by disruptions in vascular supply, intravascular blockages, or pressure on blood vessels, leading to diminished circulation. This condition predominantly affects the long-bone epiphysis in weight-bearing joints, particularly impacting the femoral head. The ongoing global health challenge posed by the novel coronavirus disease (COVID-19) has raised awareness of its diverse clinical manifestations. While pulmonary dysfunction remains a hallmark, reports of AVN of the hip have emerged in association with COVID-19 infection. Despite existing literature documenting cases of unilateral and bilateral femoral head necrosis associated with COVID-19 infection, it is noteworthy that corticosteroid use has been identified as a significant contributing factor to the development of this condition. Here, we present a case of bilateral AVN of the femoral head in a young individual linked solely to COVID-19 infection. Existing case records show only a handful of instances where COVID-19 has led to avascular necrosis, all involving either older individuals or those with notable risk factors. What sets our case apart is that the patient is young and lacks any significant risk factors. This report aims to propose a credible connection between COVID-19 infection and femoral head osteonecrosis in young patients not exposed to steroid treatment.

## Introduction

The SARS-CoV-2 virus is the causative organism behind COVID-19 infection. While most individuals infected with the virus typically present with a respiratory illness that is mild to moderate in severity and recuperate without necessitating specific medical intervention, older individuals and those with pre-existing health conditions or impaired immune systems are more prone to developing severe illnesses. In addition to respiratory symptoms, COVID-19 has been found to cause both arterial and venous thrombosis [1]. While many individuals do indeed recover from COVID-19, it can have detrimental impacts on various organ systems, often manifesting as part of the post-COVID-19 syndrome [2].

Osteonecrosis, or ischemic necrosis of the head of the femur, is a chronic, debilitating disease distinguished by a disordered pattern of cell necrosis and a multifaceted repair mechanism involving bone resorption and formation. The condition primarily stems from insufficient blood supply, which causes cell death in bone tissue, subsequently causing joint destruction and dysfunction. It is important to emphasize that the manifestations of the disease arise not solely from cell death but also from the resorptive aspect of the repair process, leading to compromised structural integrity. This further causes fractures primarily in the subchondral region. Reduced blood flow to the femoral head can occur through three processes that share a common pathophysiology: fractures or dislocation causing vascular compromise, intravascular blockage due to thrombi or embolic fat, or intraosseous extravascular compression arising from Gaucher cells or hypertrophy of lipocytes [3].

This case report outlines the details of a 29-year-old man who developed bilateral avascular necrosis of the head of the femur, possibly caused by COVID-19 infection. In the absence of disease-specific risk factors, we would like to suggest a probable association between COVID-19 infection and the development of bone osteonecrosis.

## Case presentation

**Figure 1:**
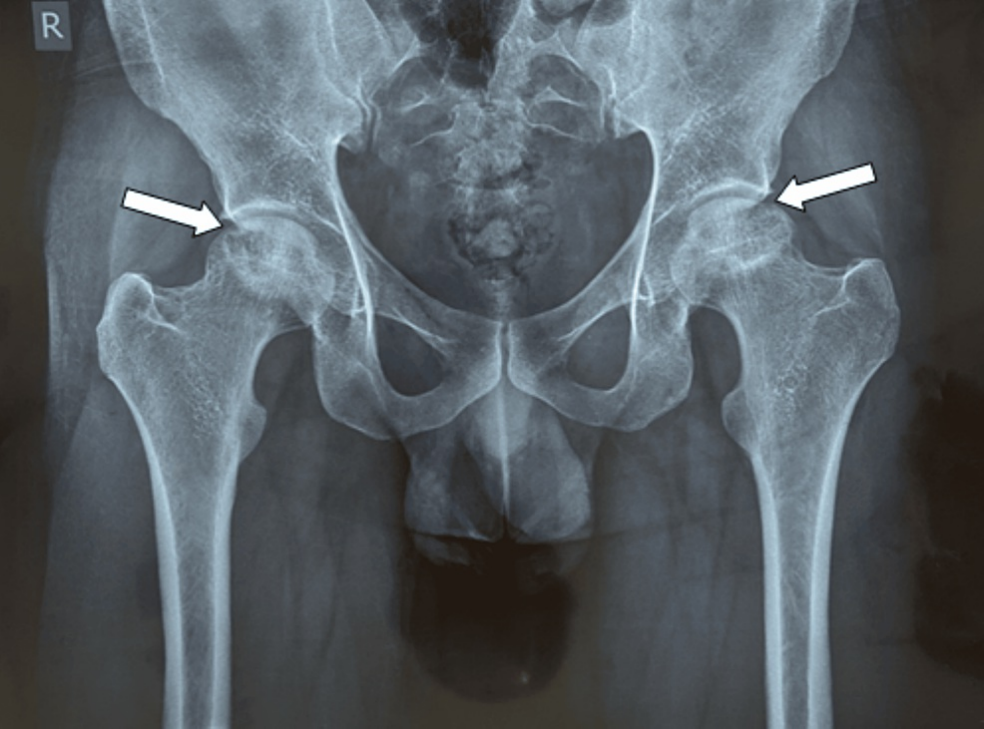
Plain X-ray bilateral hips: anteroposterior view Plain radiograph of the hip showing bilateral osteopenia and patchy sclerosis consistent with bilateral femoral osteonecrosis (marked with arrows)

**Figure 2:**
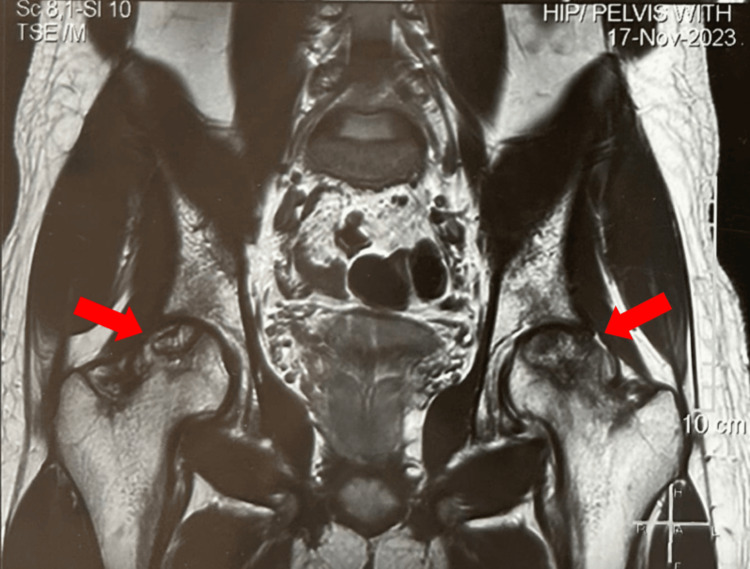
MRI bilateral hip: anteroposterior view Magnetic resonance imaging of the hips showing well-defined low signal intensity (sclerotic rim) with subtle marrow edema and minimal hip joint infusion bilaterally (indicated by arrowheads) suggesting femoral avascular necrosis

This is a case of a 29-year-old Asian male who visited the clinic in October 2023, reporting an abrupt onset of left hip pain persisting for three months, without any previous history of trauma to the joint. The pain progressively intensified, causing significant distress to the patient, and did not subside despite taking rest and using over-the-counter painkillers. He started limping to avoid bearing weight on the affected joint. His past medical history was unremarkable except for the COVID-19 infection in December 2022, which was managed conservatively. He was a non-alcoholic, non-smoker, and had not previously received any form of steroids. His family history was negative for similar illnesses and autoimmune disorders. He was prescribed Naproxen 500 mg twice daily to combat the pain. For further evaluation, a plain X-ray of the bilateral hip was ordered, which revealed osteopenia and significant sclerosis of the bilateral femoral head, indicating bilateral femoral osteonecrosis (Figure 1). To confirm the diagnosis, magnetic resonance imaging (MRI) of the pelvis and bilateral hips was done (Figure 2), which revealed evidence suggestive of ischemic necrosis of bilateral femoral heads (left > right), Stage II by the Ficat-Arlet classification (Table 1).

**Table 1:** Ficat-Arlet Classification

Stages	Features			
	Plain radiograph	Bone scan	MRI	Clinical features
Stage 0	No findings	-	No findings	None
Stage I	Findings can range from normal radiographs to minor osteopenia	Increased uptake	Edema	Pain specifically in the groin
Stage II	Mixed osteopenia and/or sclerosis and/or subchondral cysts, without any subchondral lucency (crescent sign)	Increased uptake	Geographic effect	Pain associated with stiffness in the groin
Stage III	Crescent sign and eventual cortical collapse	-	Similar to plain radiograph	Pain associated with limp and stiffness may radiate to the ipsilateral knee
Stage IV	Terminal-stage with signs of secondary degenerative alterations	-	Similar to plain radiograph	Pain with limp

He was advised to proceed with bilateral total hip replacement surgery, but the patient cited personal reasons and preferred to start physical therapy before proceeding with surgery. While his left hip pain gradually worsened and stabilized, he developed a new-onset pain in his right hip, which made it difficult for him to ambulate. To identify the potential etiological factors responsible for causing the disease, anti-nuclear antibodies, sickle cell hemoglobin S, rheumatoid factor, serum calcium level, CRP, and ESR were ordered. The findings of the aforementioned investigations are listed in Table 2. He was treated symptomatically with analgesics for pain management while awaiting total hip replacement surgery.

**Table 2:** Laboratory Investigations WBC: white blood cell; RBC: red blood cell; TIBC: total iron binding capacity; Fe: iron; CRP: C-reactive protein; ANA: antinuclear antibody; HbS: sickle hemoglobin; N: normal.

Variables	Reference Values	Value Upon Presentation	Date Taken
Calcium	8.6–10.2 mg/dL	9.7	October 13, 2023
Albumin	4.3-5.2 g/dL	4.6	October 13, 2023
WBC	3.4-10.8×10^3^/µL	6.2	October 13, 2023
RBC	4.14-5.80×10^6^/µL	4.81	October 13, 2023
Triglycerides	0-149 mg/dL	64	October 13, 2023
Total cholesterol	100-199mg/dL	124	October 13, 2023
TIBC	250-450 µg/dL	300	October 14, 2023
Fe	38-169 µg/dL	61	October 14, 2023
Fe saturation	15-55%	18	October 14, 2023
Ferritin	30-400 ng/mL	84	October 14, 2023
CRP	0.3-1 mg/dL	0.4	February 14, 2024
ANA	Negative if <1:80	Negative	February 14, 2024
Rheumatoid factor	N: <14 IU/mL	<10	February 14, 2024
HbS	N: Absent	Negative	February 14, 2024
d-Dimer	<0.5	0.02	February 14, 2024

## Discussion

Avascular necrosis is a painful bone condition resulting from compromised vascular supply leading to osseous cell death and disintegration of bone tissue. The causes of AVN include idiopathic, post-traumatic incidents (most frequently affecting the head of the femur and the humeral head of the upper arm), metabolic/genetic conditions such as sickle cell anemia, thalassemia, or Gaucher disease, medication usage (particularly steroids), and excessive alcohol consumption [4].

A thorough review of the literature reveals a possible correlation between the SARS-CoV-2 virus and the emergence of osteonecrosis. Our patient had no potential risk factors stated above except for an infection with COVID-19 eight months before the onset of symptoms. Although many cases of COVID-19 infection leading to the development of osteonecrosis could be attributable to the use of steroids in the treatment, the uniqueness of this report lies in the fact that the patient was relatively young and was managed conservatively.

The pathogenesis of avascular necrosis can be explained by various theories, as elucidated below. First, the systemic inflammatory response triggered by COVID-19 releases cytokines such as Interleukin-17 and Tumor Necrosis Factor-alpha, which in turn hinder osteoblast proliferation and maturation [5]. Second, the SARS-CoV-2 virus induces a deficiency in angiotensin-converting enzyme 2 (ACE2), which further accentuates bone loss [6, 7]. Third, systemic inflammation, coupled with direct endothelial damage caused by COVID-19, leads to upregulation of tissue factor and deregulation of the coagulation cascade, frequently resulting in a hypercoagulable condition, which increases the risk of bone necrosis [8, 9]. This finding was corroborated by certain cadaveric studies, revealing coagulopathy and intravascular thrombosis in individuals following COVID-19 infection [10]. Additionally, observations from reports on osteonecrosis of the head of the femur developing after SARS-CoV-2 infection indicate that osteonecrotic lesions typically diminish in size and stabilize over time, in contrast to ischemic necrosis that develops after steroid use in other conditions [11, 12].

Early diagnosis and prompt treatment of AVN are critical to prevent disease progression [13]. The non-invasive diagnostic modalities for AVN include Plain X-ray, MRI, bone scintigraphy, and computed tomography [14]. MRI is the gold standard imaging technique for diagnosing bone osteonecrosis as X-rays have a low sensitivity of only 41% in detecting early cases [15, 16]. If not adequately managed, the disease leads to osteoarthritis and, eventually, joint replacement [17]. The above case was confirmed to have AVN by an MRI.

Various treatment methods, both surgical and non-surgical, have been outlined for addressing AVN. During the initial stages of AVN, the primary aim of the treatment plan is to reduce the pain, enhance functionality, impede further advancement, and prevent collapse of the head of the femur. Physiotherapy, hyperbaric oxygen therapy, controlled weight-bearing, and medications such as lipid-lowering agents, vasodilators, anticoagulants, and bisphosphonates have been extensively tried to prevent further disease progression. Additionally, traditional Chinese therapies may offer potential benefits [18]. Core decompression stands out as a common treatment modality for early-stage AVN, despite criticisms regarding its efficacy [19]. Other surgical options, such as osteotomy and bone grafting, may be employed to slow disease progression. In cases where non-surgical interventions and joint-preserving techniques prove ineffective, arthroplasty emerges as a viable treatment alternative. Given the extended life expectancy of young individuals and the limited longevity of arthroplasties, preserving the head of the femur and acetabula-femoral joint remains a primary objective across treatment strategies [18].

## Conclusions

On extensive clinical evaluation, there was no clear explanation for this 29-year-old male who developed bilateral hip osteonecrosis with no traditional risk factors. A history of a positive COVID-19 infection was the only plausible inciting event. Our case report raises the prospect of a potential link between COVID-19 and the development of bone osteonecrosis in patients without comorbidities. We acknowledge that the patient could have been in a hypercoagulable state due to inflammation triggered by the virus. The AVN following COVID-19 may manifest in a variety of ways. Although the typical presentation resembles classical AVN, some patients may encounter an abrupt and severe manifestation of the disease characterized by rapid deterioration. Healthcare providers should exercise caution when managing individuals with prior COVID-19 infection. Consistent monitoring and routine assessment of hip discomfort during subsequent visits, as well as enhanced awareness, may aid in the early detection of AVN and the prevention of degenerative joint disease. Consequently, we advocate further research to explore the correlation between COVID-19 infection and AVN, especially in young individuals without any significant risk factors.
